# Clinical and Surgical Perspectives on Isolated Thoracic Ectopia Cordis: A Rare Case Report

**DOI:** 10.1155/crpe/5561918

**Published:** 2025-09-01

**Authors:** Syed Mohsin Raza Bukhari, Hassan Mehdi, Maham Zaman, Nithya Venkatesh, Ammar Abbas, Ishrat Fatima, Aqsa Farooq, Hassaan Raza, Mohsin Raza

**Affiliations:** ^1^Department of Thoracic Surgery, Nishtar Medical University and Hospital, Multan, Pakistan; ^2^Department of Thoracic Surgery, Quaid-e-Azam Medical College, Bahawalpur, Pakistan; ^3^Department of Thoracic Surgery, S.D. Asfendiyarov Kazakh National Medical University, Almaty, Kazakhstan; ^4^Department of Thoracic Surgery, Services Institute of Medical Sciences, Lahore, Pakistan; ^5^Department of Paediatrics, Fatima Jinnah Medical College, Lahore, Pakistan; ^6^Department of Thoracic Surgery, Avicenna Tajik State Medical University, Dushanbe, Tajikistan

**Keywords:** congenital heart disease, ectopia cordis, neonate, preterm delivery

## Abstract

Ectopia cordis is an exceptionally uncommon congenital condition where the heart develops outside its normal position due to incomplete closure of the ventral chest wall during embryogenesis. The anomaly may occur in isolation or with other structural defects, often resulting in a poor prognosis despite advancements in medical and surgical care. This report discusses a preterm neonate delivered at 33 weeks of gestation following an uneventful pregnancy in a dizygotic twin gestation. The neonate, diagnosed with thoracic ectopia cordis, displayed respiratory distress at birth but showed no significant cardiac or extracardiac abnormalities, a rare presentation. Echocardiography identified minor findings, including a small secundum atrial septal defect and trivial valve regurgitation, with otherwise normal cardiac structure and function. The initial management involved covering the exposed heart with sterile dressings, administration of antibiotics, and supportive care. Surgical correction to approximate the chest wall was successfully performed, but the neonate succumbed 2 days postoperatively. This case stands out due to the absence of complex anomalies typically associated with ectopia cordis and highlights the diagnostic and therapeutic challenges encountered in such rare conditions. Cases such as this contribute to a deeper understanding of ectopia cordis and reinforce the need for improved strategies to enhance survival outcomes in similar scenarios.

## 1. Introduction

Ectopia cordis, a rare congenital defect affecting 5.5–7.9 per million live births, involves the heart being positioned outside the thoracic cavity and constitutes 0.1% of all congenital heart disorders. It can appear as an isolated condition or alongside other structural abnormalities [1]. Prognosis largely depends on the size and placement of the defect, with survival chances compromised by rapid postnatal deterioration [2]. Ectopia cordis is commonly associated with cardiac and extracardiac anomalies, including ventricular septal defect (VSD), tetralogy of Fallot (TOF), atrial septal defect (ASD), central nervous system malformations, gastroschisis, and omphalocele [3]. Isolated thoracic ectopia cordis, without any intracardiac anomaly, is even rarer [4, 5].

Although the etiology of ectopia cordis remains unclear, it is often linked to chromosomal abnormalities, such as Turner syndrome and Trisomy 18, with theories suggesting the primary failure of heart descent, rupture of the chorion or yolk sac, or lack of functional bone morphogenetic protein [6, 7]. Prenatal ultrasonography screening is crucial for early diagnosis, and immediate referral to specialized care is essential for management. Despite progress in neonatal cardiac surgery, complete thoracic or thoracoabdominal ectopia cordis remains a formidable challenge, with few patients achieving long-term survival.

Ectopia cordis requires urgent stabilization and protection from infection in resource-limited settings. Categorization includes cervical to abdominal locations. Association with an omphalocele suggests the pentalogy of Cantrell. Early surgery improves outcomes, emphasizing the need for enhanced collaboration and resource allocation [8].

We present a rare case of a preterm neonate delivered at 33 weeks of gestation having thoracic ectopia cordis with no major cardiac or extracardiac abnormality.

## 2. Case Presentation

**Figure 1:**
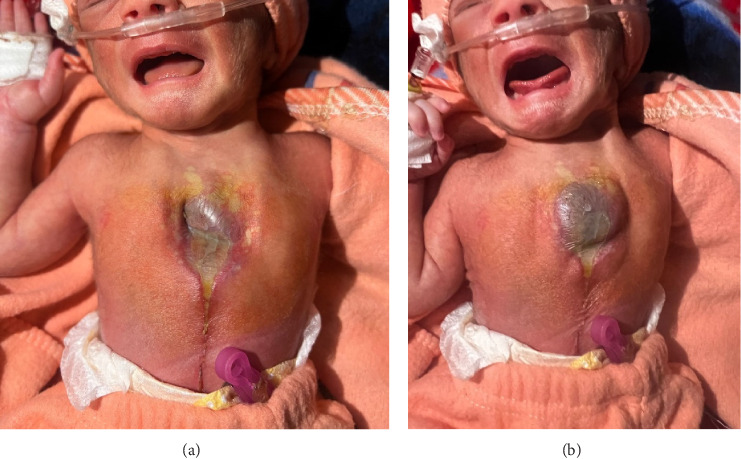
(a) A newborn with ectopia cordis. (b) Neonate with ectopia cordis in a plain view.

This case involves a 2-day-old female neonate born to a 19-year-old mother (Pashtun), Gravida 1, Para 1. The pregnancy was a complicated dizygotic, dichorionic diamniotic twin gestation (determined postnatally through placental examination), delivered preterm at 33 weeks via emergency cesarean section. Spontaneous premature rupture of membranes at 33 weeks precipitated preterm labor, necessitating an emergency C-section, and the cardiac surgeon was unavailable at the time of delivery. The mother gave birth to fraternal twins, one female and one male. Both neonates were admitted to the neonatal intensive care unit (NICU); the female was diagnosed with ectopia cordis (Figure 1), while the male had aspiration pneumonia.

The female neonate presented with respiratory distress and grunting, accompanied by a low-grade fever. Physical examination revealed the absence of the sternum, clitoral hypertrophy, and a patent anal opening. On examination, her respiratory rate was 43 breaths per minute, pulse rate 133 bpm, SpO_2_ 97% on 2.5 L of oxygen, and her weight was 1.8 kg. Lab results showed RBCs of 4.72 million/mm^3^, hemoglobin of 17.7 g/dL, platelets of 72.6 × 10^3^/μL, and hematocrit of 55%. The heart's rhythmic movements outside the chest cavity are depicted in the supporting files (Video 1). Following birth, the neonate's heart was covered with saline-soaked sterile dressings, and she received intravenous antibiotics and vitamin K injections. The neonate was referred to the cardiothoracic surgery department for further evaluation and intervention.

**Figure 2:**
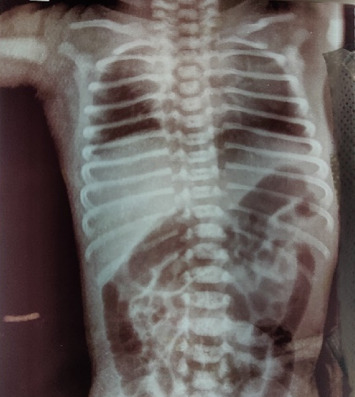
X-ray PA view showing absent sternum and collapse of the left upper lobe.

**Figure 3:**
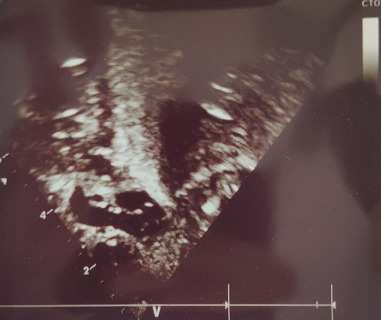
Echocardiography of a 3-day-old neonate with ectopia cordis, showing no major cardiac defects.

X-ray (Figure 2) showed an absent sternum with a collapsed left lung. The echocardiography (Figure 3) report revealed ectopia cordis with no major cardiac defect and a structurally normal heart with levocardia and atrial situs solitus, indicating proper anatomical positioning of the heart. Both atrioventricular (AV) and ventriculoarterial (VA) connections were concordant, ensuring normal blood flow between the heart chambers and the arteries. There was a notable aneurysmal fossa ovalis accompanied by a small secundum ASD, but the interventricular septum remained intact. The left ventricle demonstrated normal dimensions and function, with all heart valves appearing normal. Mild tricuspid and mitral regurgitation were present but deemed trivial, with no significant patent ductus arteriosus (PDA) identified. The aortic arch was normal, and there was no pericardial effusion, suggesting a healthy cardiac status overall.

The mother was not very compliant with her antenatal visits, attending only two or three times due to the lack of a well-equipped prenatal diagnostic center nearby. She had to travel to the city for check-ups, which made regular visits difficult. As a result, she was unaware of the condition of the neonate prenatally. According to her, the pregnancy was smooth, and no abnormalities were detected during her limited prenatal visits, possibly due to inadequate facilities. She reported no history of diabetes mellitus, hypertension, or ischemic heart disease. She denied antenatal history of fever, infection, smoking, or teratogenic exposures. She took folic acid, calcium, and iron supplements, and her maternal immunization was complete. Although there was no family history of similar cases, there is a positive history of consanguinity, as her husband is a first-degree relative.

**Figure 4:**
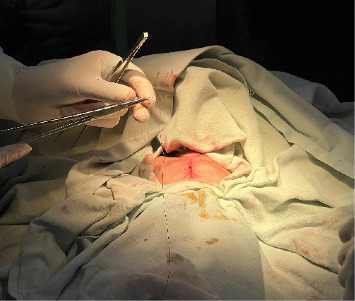
Chest wall approximation.

**Figure 5:**
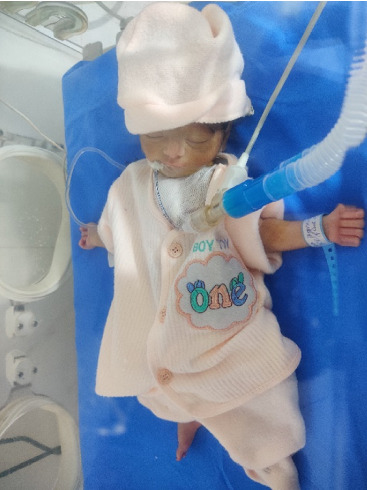
Neonate in an incubator in the neonatal intensive care unit (NICU) after surgery.

The patient underwent cardiothoracic surgery involving an approximation of the chest wall (Figure 4) under general anesthesia, which was successful. Intraoperative chest wall approximation is shown in the supporting files (Video 2). After the operation, she was shifted to the NICU and placed in an incubator (Figure 5). The newborn expired 2 days after undergoing surgery. The parents were informed, and counseling was provided related to marriage to a first-degree relative.

## 3. Discussion

Ectopia cordis is a birth abnormality characterized by the placement of the heart outside the thorax. Its prevalence is 0.1% of all cardiac abnormalities. It may occur alone or in conjunction with other cardiac abnormalities. In the present case, a preterm neonate diagnosed with thoracic ectopia cordis was delivered at 33 weeks of gestation. This condition is remarkable due to the absence of significant cardiac and extracardiac abnormalities, unlike the more common associations such as gastroschisis and omphalocele [3].

Ectopia cordis is caused by improper ventral body wall (chest) construction and midline mesoderm maturation during embryonic development [9]. Although the precise cause is yet unknown, anomalies in the lateral folds of the body wall are thought to be implicated. The ventral wall is often formed by the merging of the lateral body walls at the midline. Ectopia cordis may be caused by the corruption of this process [10]. In our case, the neonate also exhibited clitoral hypertrophy and a patent anal opening, highlighting the spectrum of associated anomalies. A heart without the pericardium, sternum, or skin to protect it is the result of defective ventral body wall construction. It is also possible that organs other than the skin developed. Congenital cardiac abnormalities, in which the heart has not formed correctly, are linked to many cases of ectopia cordis.

Ectopia cordis is associated with various genetic and environmental factors. Chromosomal abnormalities, particularly Trisomy 13 (Patau syndrome) and other aneuploidies, have been linked to this condition [6]. In addition, specific single-gene disorders that affect cardiac development are implicated, with mutations in genes such as NKX2-5, TBX5, and GATA4 playing significant roles. Some cases are also associated with mutations in the Sonic hedgehog (SHH) gene, which is crucial for the development of midline structures in the body. Ectopia cordis can occur as part of broader syndromes, most notably the Pentalogy of Cantrell, which includes additional defects such as diaphragmatic hernia and omphalocele. Other syndromic associations include Turner syndrome, among others. Furthermore, while not strictly genetic, environmental factors such as teratogenic exposures during pregnancy, such as certain medications, alcohol consumption, or infections, may interact with genetic predispositions to heighten the risk of developing ectopia cordis. Understanding these multifactorial influences is essential for diagnosis, management, and genetic counseling for affected families [11]. However, in this case, the absence of syndromic features, normal antenatal ultrasound findings, and positive consanguinity history suggests an isolated presentation rather than a syndromic association, such as the pentalogy of Cantrell.

The primary sign of ectopia cordis is the heart's exodus from the body. Although not observed in our case, infants with this syndrome frequently exhibit additional midline defects, which are congenital anomalies occurring along the body's median axis, extending from the cranial region to the groin. These defects may include cranial clefts, orofacial clefts such as cleft lip and palate, pulmonary hypoplasia, scoliosis, and incomplete diaphragmatic fusion [12].

Ectopia cordis is classified into cervical, thoracic, thoracoabdominal, and abdominal types [13]. Thoracic ectopia cordis, as in this case, generally carries a poor prognosis. As many as 80.2% of people with ectopia cordis have associated intracardiac defects, such as VSD (100%), ASD (53%), TOF (20%), left ventricular diverticulum (LVD, 20%), and pulmonary hypoplasia [6]. In this case of thoracic ectopia cordis, the neonate displayed respiratory distress, with tachypnea and elevated hematocrit levels, potentially indicating underlying hypoxia. The echocardiographic evaluation revealed a structurally normal heart, apart from a small secundum ASD and trivial valve regurgitation, further emphasizing the rarity of an isolated ectopia cordis case without significant cardiac anomalies.

Prenatal ultrasonography is important for early diagnosis of ectopia cordis, with cases diagnosed as early as 9 weeks' gestation. In minor forms, diagnosis through ultrasound can be difficult; however, three-dimensional scanning provides better results in such cases [14, 15]. In this case, the missed antenatal diagnosis was more likely due to limitations in early ultrasound screening rather than operator error. Although this case lacked prenatal diagnosis due to limited imaging resources, in current clinical settings, ectopia cordis and associated anomalies of pentalogy of Cantrell can be detected as early as the first trimester using high-resolution ultrasound or fetal echocardiography. Early diagnosis allows in utero referral to tertiary centers with pediatric cardiac surgery services, improving neonatal outcomes through immediate postnatal intervention. According to Pośpiech-Gąsior et al. in a review of 57 cases diagnosed prenatally, approximately 18% of fetuses survived postnatally, demonstrating that early detection and perinatal planning can significantly alter expected outcomes [16]. In our case, the neonate was delivered at a local hospital, transferred by car to a tertiary care center for further management. Failure to detect the anomaly prenatally precluded timely in utero transfer, which may have impacted the clinical course.

Early diagnosis and termination of pregnancy are often considered the mainstay solutions in many cases of ectopia cordis. However, some case reports have documented patient survival following surgical correction [17]. One case report described a child with ectopia cordis who survived for 6 years without any surgical intervention [18]. The surgical procedures used for the correction of ectopia cordis include thoracoplasty and the use of mandibular distractors [19, 20].

The patient underwent chest wall repair surgery, but survival was limited to 2 days postoperatively, reflecting the inherent challenges of managing such severe defects despite optimal intervention. This underscores the importance of early diagnosis, multidisciplinary care, and ongoing research to improve outcomes for this complex condition.

## 4. Conclusion

Ectopia cordis carries a high mortality rate due to limited treatment options; therefore, understanding its developmental biology and genetic basis and improving prenatal diagnostic techniques are crucial for early diagnosis and intervention. Due to its rarity, cases of ectopia cordis contribute significantly to research, benefiting both clinical practice and genetic counseling.

## Data Availability

The data supporting the findings of this study are available within the article. Further data that support the findings of this study are available from the corresponding author upon request.

## Supporting Information

A video of the newborn following birth, showcasing the movement of the heartbeat outside the thoracic cavity, along with footage of the surgical procedure, has been provided as a zip file in the supporting information.
